# Unravelling the prognostic and operative role of intratumoural microbiota in non‐small cell lung cancer: Insights from *16S rRNA* and RNA sequencing

**DOI:** 10.1002/ctm2.70156

**Published:** 2025-01-03

**Authors:** Fuling Mao, Zixuan Hu, Ruifeng Shi, Hongbing Zhang, Zihe Zhang, Yongwen Li, Xuanguang Li, Penghu Gao, Jinhui Li, Minghui Liu, Hongyu Liu, Jun Chen

**Affiliations:** ^1^ Department of Lung Cancer Surgery Tianjin Medical University General Hospital Tianjin China; ^2^ Department of Thoracic Surgery and Oncology the First Affiliated Hospital of Guangzhou Medical University State Key Laboratory of Respiratory Disease National Clinical Research Center for Respiratory Disease Guangzhou Institute of Respiratory Health Guangzhou China; ^3^ Tianjin Key Laboratory of Lung Cancer Metastasis and Tumor Microenvironment Tianjin Lung Cancer Institute Tianjin Medical University General Hospital Tianjin China

**Keywords:** microbiota, non‐small cell lung cancer (NSCLC), *Peptococcus*, prognosis, protective and harmful microbial clusters

## Abstract

**Background:**

Complex interrelationships between the microbiota and cancer have been identified by several studies. However, despite delineating microbial composition in non‐small cell lung cancer (NSCLC), key pathogenic microbiota and their underlying mechanisms remain unclear.

**Methods:**

We performed *16S rRNA* V3–V4 amplicon and transcriptome sequencing on cancerous and adjacent normal tissue samples from 30 patients with NSCLC, from which clinical characteristics and prognosis outcomes were collected. We used *16S rRNA* sequencing to dissect microbial composition and perform prognosis correlations, and in conjunction with transcriptome sequencing, we determined potential mechanisms underpinning significant microbiota actions.

**Results:**

In comparing different sample types, we identified more pronounced beta diversity disparity between NSCLC, lung squamous cell carcinoma (LUSC) and corresponding paired normal tissues. Concurrently, LUSC and lung adenocarcinoma exhibited distinct microbial composition traits at genus levels. Subsequently, four phyla, five classes, nine orders, 17 families and 36 genera were filtered out and were related to prognosis outcomes. Intriguingly, a protective microbial cluster was identified encompassing nine genera associated with delayed disease recurrence, with functional analyses suggested that these microbiota predominantly exerted metabolism‐related functions. Additionally, a harmful microbial cluster (HMC) was identified, including three genera. In this HMC and subsequent prognosis model analyses, harmful intratumoural microbiota were potentially implicated in infection, inflammation and immune regulation. Crucially, we identified a microbial genus, *Peptococcus*, which was as an independent, detrimental NSCLC prognostic factor and potentially impacted prognosis outcomes via tumour necrosis factor (TNF) signalling.

**Conclusions:**

We identified a substantial connection between intratumoural microbiota and NSCLC prognosis outcomes. Protective microbiota primarily exerted metabolic functions, whereas harmful microbiota were mainly implicated in infection, inflammation and immune modulation. Furthermore, *Peptococcus* may be significant in adverse NSCLC prognoses and serve as a potential biomarker for patient management and cancer screening.

**Key points:**

- Four phyla, five classes, nine orders, 17 families and 36 genera have been found associated with NSCLC prognosis.
- We identified a protective microbial cluster associated with delayed recurrence and a harmful microbial cluster related to shorter survival and earlier recurrence.
- We identified *Peptococcus* as an independent, detrimental prognostic factor for NSCLC, potentially impacting prognosis via TNF signalling.

## Graphical abstract

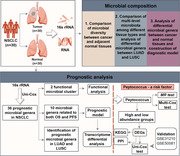

## 1 BACKGROUND

Lung cancer is a significant public health issue and represents the foremost cause of tumour‐related mortality at a global level. Specifically, non‐small cell lung cancer (NSCLC) comprises approximately 80–85% of all lung cancer instances.^1^, ^2^ NSCLC incidence and mortality rates in China are also considerable, making it a devastating disease with a profound impact on public health.^2^ The most prevalent histological NSCLC subtype is lung adenocarcinoma (LUAD), while lung squamous cell carcinoma (LUSC) ranks second. Globally, LUAD has become increasingly prevalent and gradually shown higher incidence trends among women.^2^ Therefore, molecular strategies preventing and treating lung cancer must be identified to address this major health challenge.

In recent years, correlations between the microbiota and different diseases have been comprehensively studied.^3^ Concomitant with extensive tumour exploration, the notion that tumours are sterile environments has been abandoned, with microbiota species identified across several cancers.^4^ Moreover, the microbiota may be indispensable tumour cell and tumour microenvironment (TME) constituents.^3^, ^4^, ^5^

Many studies have reported that intratumoural microbiota may be involved in heterogeneous processes, such as tumourigenesis, progression, metastasis and different treatments.^5^, ^6^, ^7^ Currently, many microbiota species are believed to be strongly associated with tumourigenesis, including *Helicobacter pylori* in gastric cancer,^8^ *Fusobacterium nucleatum* in colorectal cancer (CRC),^9^ and human papillomavirus in cervical cancer.^10^ In contrast, safeguarding probiotics have also been identified in tumours, with studies reporting that *Akkermansia muciniphila* may restrain tumour growth via diverse pathways, thereby activating apoptosis, modulating the tumour immune microenvironment (TIME) or generating specific metabolites.^11^, ^12^, ^13^

Although many studies have reported a nexus between the microbiota and tumours, associations between the microbiota and NSCLC remain unclear^3^; specifically, no investigations have examined the microbiota pertaining to NSCLC prognoses in patients. Therefore, in this study, we screened for prognostic microbiota in NSCLC and explored underlying molecular mechanisms.

We performed *16S rRNA* and transcriptome sequencing on cancerous and adjacent normal tissues from 30 patients with NSCLC, from which clinical and prognostic information was recorded. Our remit was to pinpoint the key microbiota influencing NSCLC prognosis outcomes in these patients and provide a platform for patient management and cancer screening.

## 2 METHODS

### 2.1 Study design and sample collection

Between January 2018 and December 2019 at the Department of Lung Cancer Surgery, Tianjin Medical University General Hospital, we collected cancerous and adjacent normal tissues from 30 consenting patients with primary NSCLC who underwent surgical resection for primary lung cancer.

Inclusion criteria were as follows: (1) a definitive, primary pathological NSCLC diagnosis; (2) patients in the 30–80 year old age range; (3) patients not receiving any anti‐tumour therapy, such as chemotherapy, targeted therapy, immunotherapy or radiotherapy; (4) patients showing no traces of acute, exacerbated chronic obstructive pulmonary disease, complex pneumonia, infectious bronchiectasis, acute asthma or bronchitis, no purulent or fever or grey phlegm and no other malignant diseases; (5) patients with no history of hormone use and antibiotic‐related treatments at least 1 month before surgery.

Tissue samples were aseptically collected at surgery, transferred to cryovials, promptly snap‐frozen in liquid nitrogen and stored in the Tianjin Lung Cancer Institute. Matched adjacent normal tissue samples were situated at >5 cm from the tumour tissue edge. Overall survival (OS) was defined as the period extending from surgery to death or the last follow‐up time for patients who remained alive. Progression‐free survival (PFS) was the time from surgery to disease progression or death resulting from any cause.

### 2.2 *16S rRNA* sequencing

Microbiota gDNA was extracted from tissue samples. Polymerase chain reaction (PCR) was performed using primers targeting the V3–V4 region of *16S rRNA* (338F: 5′‐ACTCCTACGGGAGGCAGCAG‐3′ and 806R: 5′‐GGACTACHVGGGTWTCTAAT‐3′). After purification and quality assessment, final products were sequenced on an Ion S5™ XL platform (Thermo Fisher Scientific, USA). Clean data were generated from the preliminary quality control of raw data (removing barcodes, primers and chimeras, and splicing and filtering low‐quality sequences). Uparse software was used to cluster clean reads into operational taxonomic units (OTUs) with 97% identity. Mothur software and the SILVA132 SSUrRNA database were used for OTU annotation analysis.^14^, ^15^

### 2.3 Transcriptome sequencing

All samples underwent RNA extraction in accordance with manufacturer's protocols of Trizol (Invitrogen, Carlsbad, California, USA). RNA quantity and quality were measured by gel electrophoresis and a NanoPhotometer spectrophotometer (Implen GmbH, Munich, Germany). Total RNA quantity for library construction was not <1 µg. Next, using the NEBNext UltraTM RNA Library Prep Kit (New England Biolabs, New Jersey, USA), and after mRNA enrichment, fragmentation processing, reverse transcription to cDNA, adapter addition, PCR amplification and product purification, libraries were generated. After insert size quantification and examination, libraries were sequenced on the Illumina sequencing platform (Illumina, California, USA). After filtering, verifying sequencing error rates and inspecting GC content, clean reads were acquired for analyses. Subread (version 1.5.0, https://subread.sourceforge.net/) was used for quantitative analysis. Reads with mapping quality values lower than 10, reads from non‐paired alignments and reads mapped to multiple regions of the genome were filtered out respectively. Finally, an original read count expression matrix was generated for bioinformatics analysis.

### 2.4 Comparison of microbial diversity

The Chao1 and Shannon indices are used to evaluate alpha diversity. Permutational multivariate analysis of variance tests were used to compared beta diversity (Bray–Curtis distances) across tissue samples. Principal co‐ordinates analysis was used for the visualisation of beta diversity.

### 2.5 Constructing a diagnosis model

DEseq2 was used to perform differential genus analysis on NSCLC and its subtypes (LUAD and LUSC) and matched normal tissues. A generalised linear model was used to calculate if a single or microbial model could discriminate between tumour and normal tissues. The pROC R package was used to demonstrate diagnostic model accuracy.

### 2.6 Screening prognosis‐related microbiota

Univariate Cox regression analysis was used to identify microbiota that were associated with OS and PFS (threshold; *p* < .05). Forest plots were also generated to show hazard ratios (HR) for prognosis‐related microbiota. Venn diagrams were also used to analyse any compositional interrelationships between microbiota related to OS and PFS.

### 2.7 Microbial ecological network analyses

An integrated network analysis pipeline was used to analyse microbial ecological networks.^16^ The sparse correlations for compositional data (SparCC) method was adopted for microbial ecological network analyses.^17^, ^18^ The parameters of SparCC are as follows: (1) The number of inference iterations to average over was 20; (2) the number of exclusion iterations to remove strongly correlated pairs was 10; (3) the number of shuffled times was 100, and the two‐sided *p* value was calculated. Cytoscape (v.3.9.1) was used to generate a final network diagram.^19^

### 2.8 Constructing a prognosis model

OTU counts were normalised using log_2_ (counts + 1), and a LASSO‐Cox regression approach to construct a microbiota‐related prognosis model based on OS using the ‘glmnet’ R package and to also predict PFS. A penalty parameter (*λ*) value was decided in accordance with the minimum partial likelihood deviation. The lambda.1se was 0.2586844 and the lambda.min was 0.1624613 in LASSO regression analyses. Risk scores were calculated as follows: risk score = sum (each genus count × corresponding coefficient). Kaplan–Meier survival curves and receiver operating characteristic (ROC) analyses were conducted to test prognosis model accuracy. In *Peptococcus* analyses for prognosis outcomes, GSE31210 and GSE50081 datasets were used to verify relationships between *Peptococcus*‐related genes and prognosis.^20^, ^21^

### 2.9 Mendelian randomisation

Summary‐level data from genome‐wide association studies (GWAS) associated with the gut microbiota were retrieved from the MiBioGen website (https://mibiogen.gcc.rug.nl/).^22^, ^23^ The studies encompassed 24 cohorts consisting of 18 340 participants and 211 taxa (131 genera, 35 families, 20 orders, 16 classes and nine phyla). Summary‐level GWAS data associated with lung cancer (ieu‐a‐a966), LUAD (ieu‐a‐a965) and LUSC (ieu‐a‐a967) were sourced from the International Lung Cancer Consortium, which is an international organisation for lung cancer researchers.^24^ We used all summary data from published studies and publicly available GWAS abstracts; therefore, no additional ethical approval or consent was required. To satisfy the three core Mendelian randomisation (MR) assumptions: relevance assumption (the genetic variant is associated with the exposure), independence assumption (the genetic variant is independent of confounding factors that affect the relationship between the exposure and outcome) and exclusion restriction assumption (the genetic variant affects the outcome only through the exposure), we conducted the following single nucleotide polymorphism (SNP) screen associated with exposure: (1) *p* value < 1e−5; (2) SNP removal from the major histocompatibility region; (3) linkage disequilibrium analysis (*r* ^2^ < 0.001, kb = 10 000); (4) SNP removal with outcome *p* values < .05; (5) SNP removal associated with confounders (PhenoScanner); (6) the *F* statistic was calculated using: *F* = $\frac{\beta^{2}}{se^{2}},$ ^25^ and SNPs were removed at *F* < 10; and (7) SNPs with palindromic structures were removed. Finally, we obtained 12 SNPs for MR analysis. In MR exposure (*Peptococcus*) and outcome analysis (lung cancer), ‘TwoSampleMR’ and ‘MRPRESSO’ R packages were used.^26^ Inverse variance weighted analysis was the primary analysis method. Weighted median and PRESSO were validation methods. Result sensitivity and heterogeneity were also analysed. Reverse analysis conditions were the same as for forward MR analysis.

### 2.10 Statistical analysis

*T*‐tests were used to analyse statistical variance between groups for which specific methods were not delineated. The Pearson method was used for correlation analysis between the microbiota and genes. The edgeR R package was used to identify differentially expressed genes (DEGs). The algorithm genewise negative binomial generalised linear model with quasi‐likelihood tests was also used. All statistical tests were two‐tailed, and statistical significance was determined at *p* < .05. The false discovery rate (FDR), used as a sole method, was used to adjust *p* values. R (v.4.3.2) was used to conduct analyses. Non‐significant (NS) values were indicated by *p* > .05, * indicated .01 < *p* < .05, ** indicated .001 < *p* < .01 and *** indicated *p* < .001.

## 3 RESULTS

### 3.1 Population demographics and clinical characteristics

We gathered 30 cancerous and adjacent normal tissue pairs from primary NSCLC patients, encompassing 17 LUAD and 13 LUSC patients. Of these, 23 were male and seven were female. The median age was 64 years and the age range was 46–83 years. Participants predominantly had stages II–IV NSCLC, with 19 lymph node metastasis and 19 smoker cases. At final follow‐up, 16 patients experienced relapse (53.3%) and 14 died (33.3%). The median OS was 952 days, while the median PFS was 526.5 days. Patient clinical characteristics are shown (Tables S1 and S2).

### 3.2 *16S rRNA* and RNA sequencing

From *16S rRNA* sequencing, the average number of reads from tumour and adjacent normal tissue samples was 77 192 and 76 981, respectively. All effective sample tags were clustered into OTUs with 97% identity, after which 10 665 OTUs were acquired. After annotation and screening, 1022 genera underwent bioinformatics analysis. From transcriptome sequencing, average raw counts numbered 91 193 198, and average clean counts after filtering numbered 88 007 549. Finally, 57 299 RNAs were annotated and used in analyses.

### 3.3 Distinct microbial composition in cancerous tissues when compared with normal tissues

**FIGURE 1:**
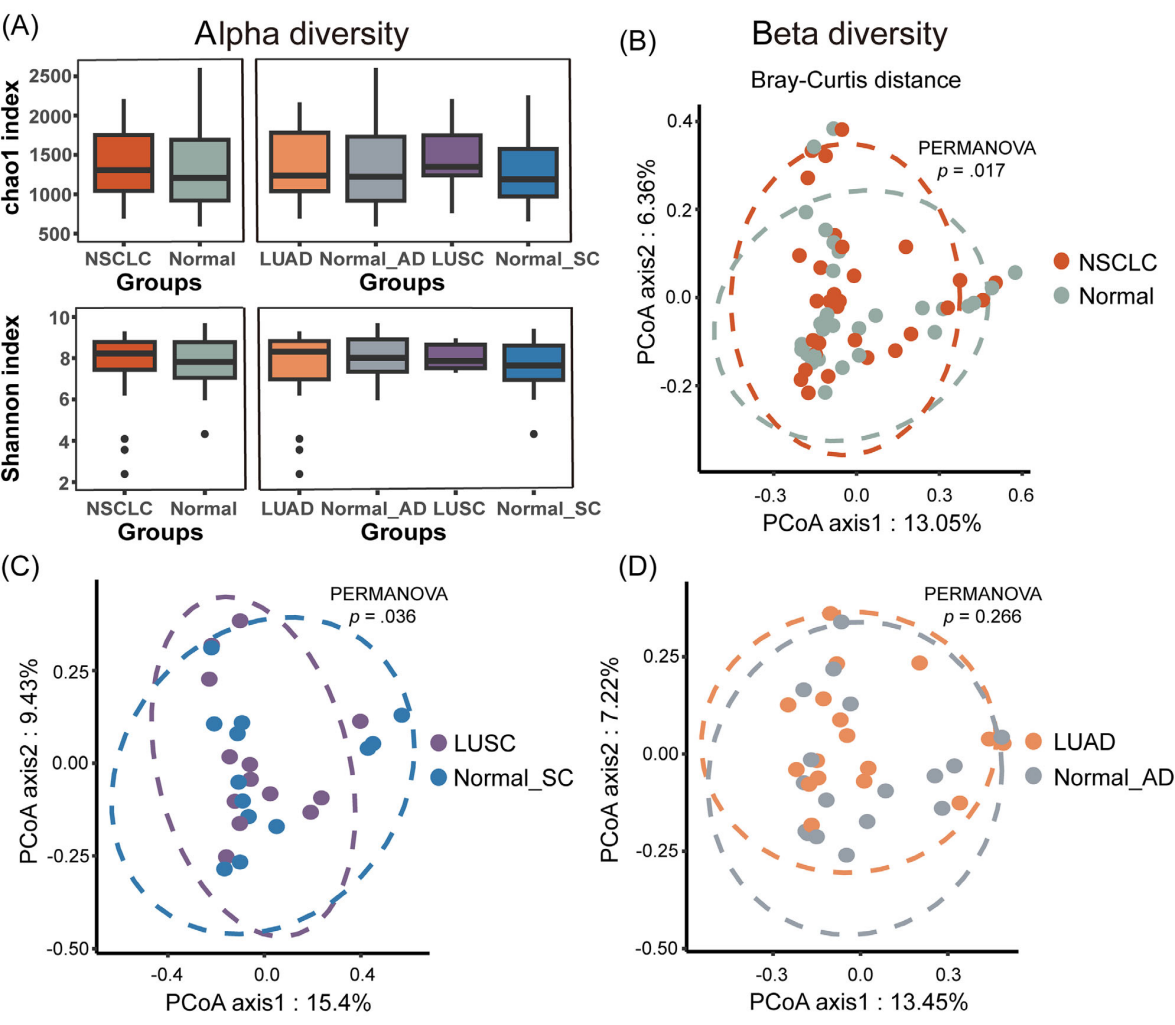
Comparing microbial diversity between cancerous and normal tissues. (A) Based on Chao1 and Shannon indices, alpha diversity comparisons were conducted between NSCLC, LUAD, LUSC and paired normal tissue samples. (B–D) Permutational multivariate analysis of variance tests (PERMANOVA) were used to compared beta diversity (Bray–Curtis distances) across tissue samples. Principal co‐ordinates analysis was used for the visualisation of data dimensionality reduction analysis. NSCLC, non‐small cell lung cancer; LUAD, lung adenocarcinoma; LUSC, lung squamous cell carcinoma; normal, normal tissues of NSCLC; normal_AD, normal tissues of LUAD; normal_SC, normal tissues of LUSC.

Alpha diversity was higher in cancerous tissues (NSCLC, LUSC and LUAD) when compared with matched normal tissues, irrespective of Chao1 or Shannon indices. This difference was not statistically significant and was probably due to low sample size. Intriguingly, the Chao1 index of LUSC samples tends to be higher than that of LUAD samples, while the trend of the Shannon index is the opposite (Figure 1A).

We next analysed beta diversity. Statistically significant differences were observed between NSCLC and matched normal tissues (*p* = .017; Figure 1B). After differentiating subtypes, we observed beta diversity differences between LUSC and matched normal tissues (LUSC vs. normal_SC: *p* = .036; Figure 1C). However, no diversity disparities were identified for LUAD (LUAD vs. normal_AD: *p* = .266; Figure 1D). Therefore, in contrast to normal tissues, LUSC tissues had a more distinctive microbial composition.

### 3.4 Microbiota comparisons across tissue types

**FIGURE 2:**
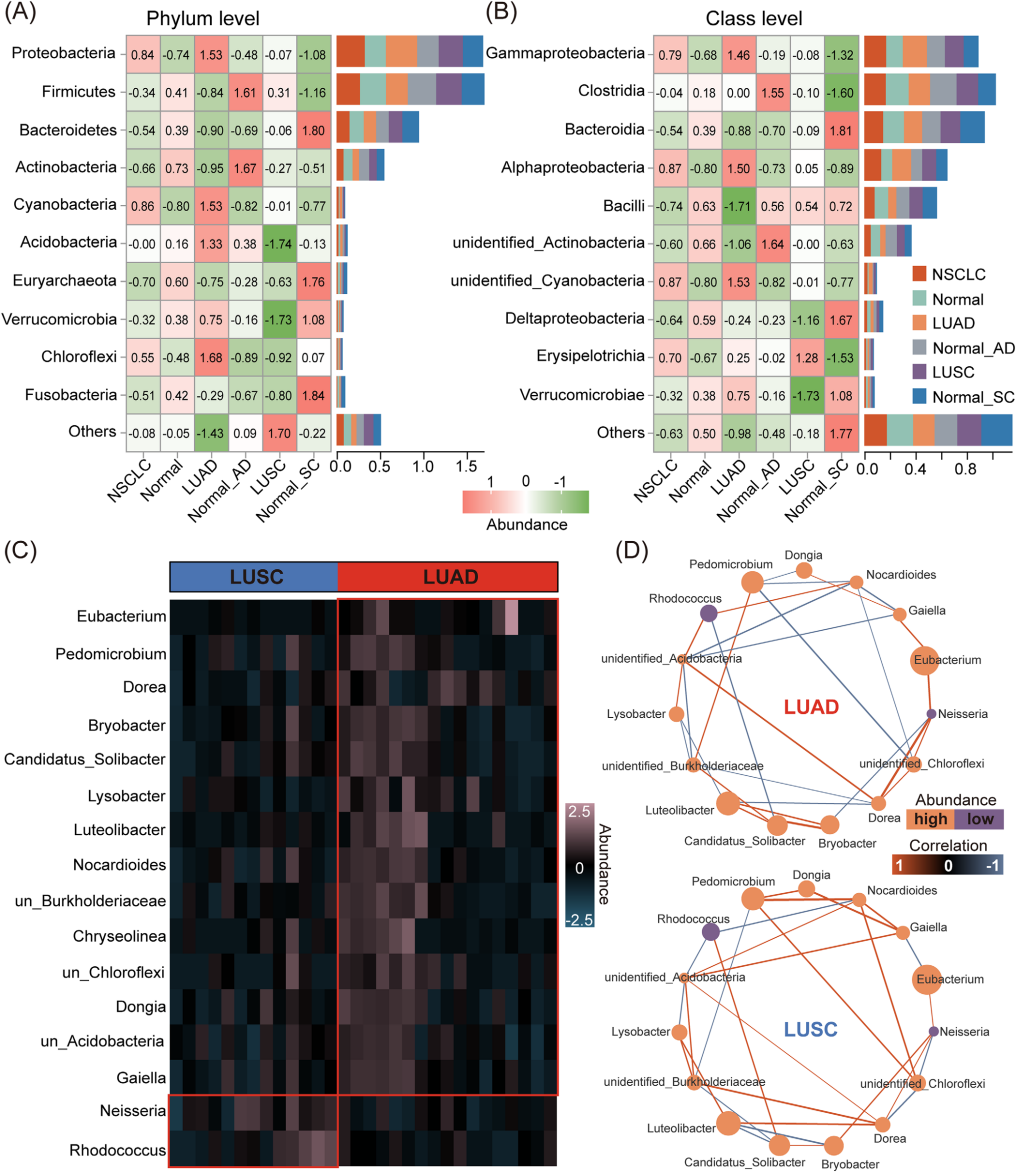
Comparing microbial composition across different tissue types. (A and B) Compositional microbiota discrepancies at phylum and class levels in different tissue types. The top 10 microbiota at each classification level were designated as high‐abundance microbiota and the remainder as low‐abundance microbiota. Values in the heatmap (left panel) are abundance values normalised by row for the purpose of comparing the differences in the abundance of each microbiota among different tissue types. A barplot (right panel) shows the abundance proportion of these microbiota in each single tissue type. (C) Microbial genera with differential abundances are shown between LUAD and LUSC (*p* < .01; |logFC| > 1) (DESeq2). (D) The ecological network shows the differences in correlations between differential microbial genera in LUAD and LUSC (SparCC).

To further determine microbial composition, we listed high‐abundance microbiota (HAM) (top 10 in average rankings) at phylum, class, order and family levels, and also low‐abundance microbiota (LAM) (others) across NSCLC histology types. HAM composition at the four levels was completely different across tissue types. At the phylum level, *Proteobacteria*, *Cyanobacteria*, *Acidobacteria* and *Chloroflexi* in LUAD were all higher than in other types but total LAM levels in LUAD were the lowest. Conversely, *Acidobacteria* and *Verrucomicrobia* in LUSC were substantially lower than in other types, but total LAM levels were the highest (Figure 2A). Unidentified microbiota were regarded as non‐important research subjects.

At the class level, eight HAM classes mainly originated from four phyla: *Proteobacteria* (including *Gammaproteobacteria*, *Alphaproteobacteria* and *DeltaproteobacteriaI* classes), *Firmicutes* (including *Clostridia*, *Bacilli* and *Erysipelotrichia* classes), *Bacteroidetes* (including the *Bacteroidia* class) and *Verrucomicrobia* (including the *Verrucomicrobiae* class). Moreover, when compared with other types, *Gammaproteobacteria* and *Alphaproteobacteria* classes and the *Proteobacteria* phylum to which the two classes belonged were both the most abundant in LUAD (Figure 2B).

By comprehensively analysing microbial composition at these four levels, ‘Other’ levels in LUAD were consistently lower across tissue types (Figure S1). To further examine microbial composition differences between LUAD and LUSC samples, we identified 19 differential microbial genera (*p* < .01). The top 16 genera are shown, of which 14 were highly abundant in LUAD and two in LUSC (Figure 2C). Our microbial ecological network also showed that correlations for these differential genera in LUAD and LUSC were significantly different (Figure 2D). This indicates that the interconnections of differential microbial genera in LUAD and LUSC are different. Therefore, we believe that LUAD microbial composition is dissimilar to LUSC.

### 3.5 Differential microbial genera analyses between cancerous and normal tissues

**FIGURE 3:**
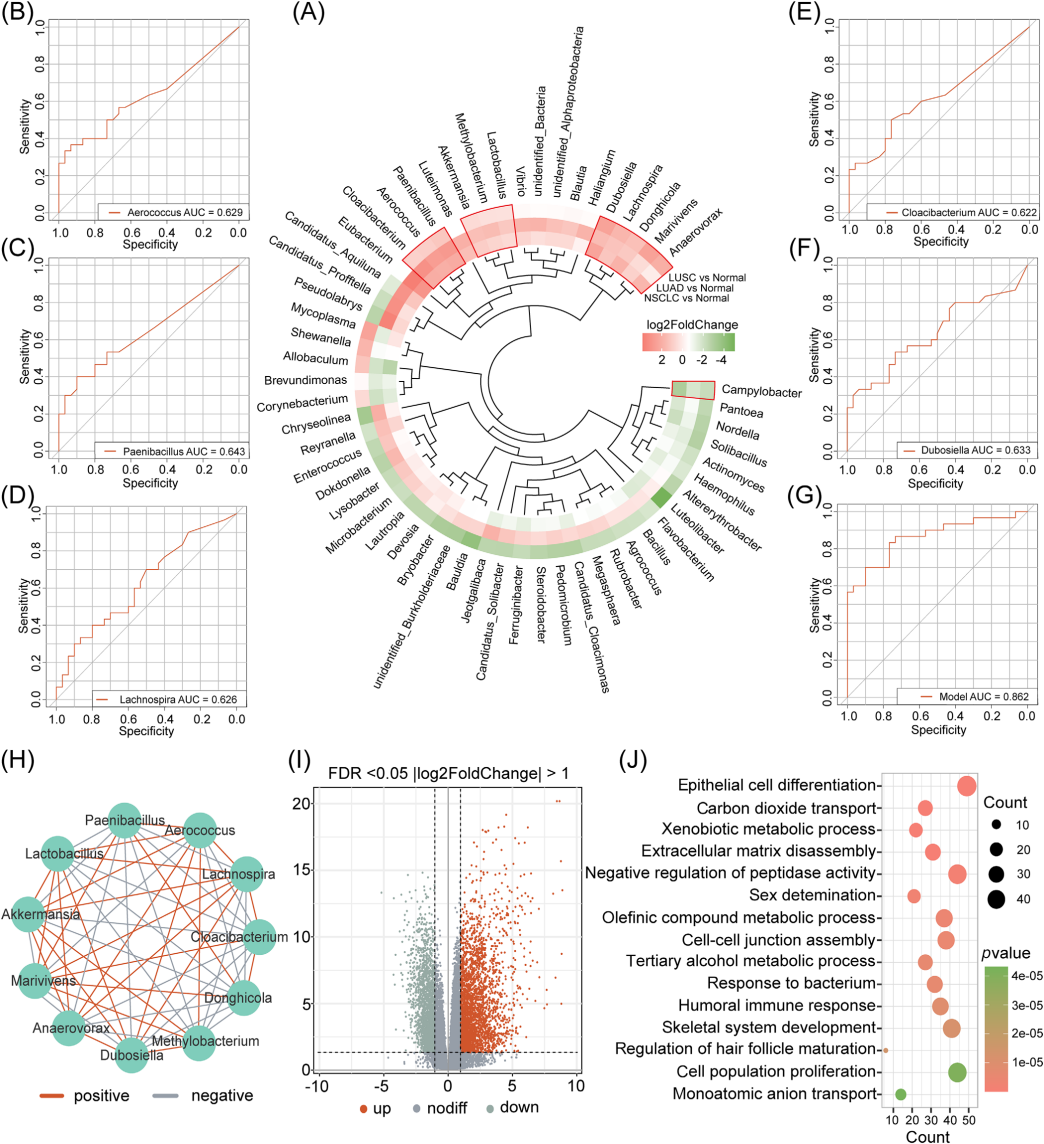
Differential microbial genera between cancer and normal tissues. (A) Microbial genera differences between NSCLC, LUSC, LUAD and paired adjacent normal tissues. A total of 12 consistently differential microbial genera in three comparisons (NSCLC vs. normal, LUSC vs. normal_SC, LUAD vs. normal_AD) were regarded as crucial differential microbial genera for subsequent construction of the diagnostic model. (B–F) The area under the curve (AUC) value in receiver operating characteristic (ROC) analysis of five crucial, differential microbial genera was 0.6. (G) The AUC value for the NSCLC diagnostic model (composed of five genera) was 0.862. (H) Ecological network analysis of 11 intratumourally highly enriched genera (SparCC, correlation coefficient > 0.4). (I) Volcano plots showing DEGs between cancer and adjacent normal tissues (FDR < 0.05; |log2FC| > 1). Red represents high gene expression in the tumour, grey represents no gene expression differences between two groups and green represents low gene expression in the tumour. (J) KEGG pathway enrichment analysis of DEGs related to intratumourally highly enriched microbial genera (FDR < 0.05; correlation coefficient > 0.4).

We also conducted microbial genus analyses between NSCLC, LUAD, LUSC and normal tissues. Ultimately, 56 genera showed abundance disparities, of which 12 displayed significant differences (differences were present across all types) between cancer and normal tissues. These consisted of 11 HAM genera in tumour (*Anaerovorax*, *Marivivens*, *Donghicola*, *Lachnospira*, *Dubosiella*, *Lactobacillus*, *Methylobacterium*, *Akkermansia*, *Paenibacillus*, *Aerococcus* and *Cloacibacterium*) and 1 HAM genera in normal tissue (*Campylobacter*, *p* < .05; Figure 3A). We next assessed genera accuracy in differentiating NSCLC and normal tissues. Critically, area under the curve (AUC) values for five genera exceeded 0.6, including *Aerococcus* (AUC = 0.629), *Paenibacillus* (AUC = 0.643), *Lachnospira* (AUC = 0.626), *Cloacibacterium* (AUC = 0.622) and *Dubosiella* (AUC = 0.633; Figure 3B–F). Any individual genus is unable to accurately predict whether the sample is NSCLC tumour tissue or normal benign tissue. From these observations, we have attempted to provide a microbial NSCLC diagnostic model (Table S3), which had a diagnostic accuracy = 0.862 (Figure 3G). This model still requires validation by more cohorts in the future.

To understand the roles of 11 HAM genera in NSCLC, we performed correlation and functional analyses. Our ecological network showed complex correlations among the 11 genera, but no closely related clusters were observed (Figure 3H). We then integrated differential expression and correlation analyses (Figure 3I) and identified 580 genera‐related genes. From Kyoto Encyclopedia of Genes and Genomes (KEGG) analysis, these aforementioned microbiota were potentially implicated in epithelial cell differentiation and metabolism‐related functions (Figure 3J).

### 3.6 The identification of prognosis‐related microbiota

**FIGURE 4:**
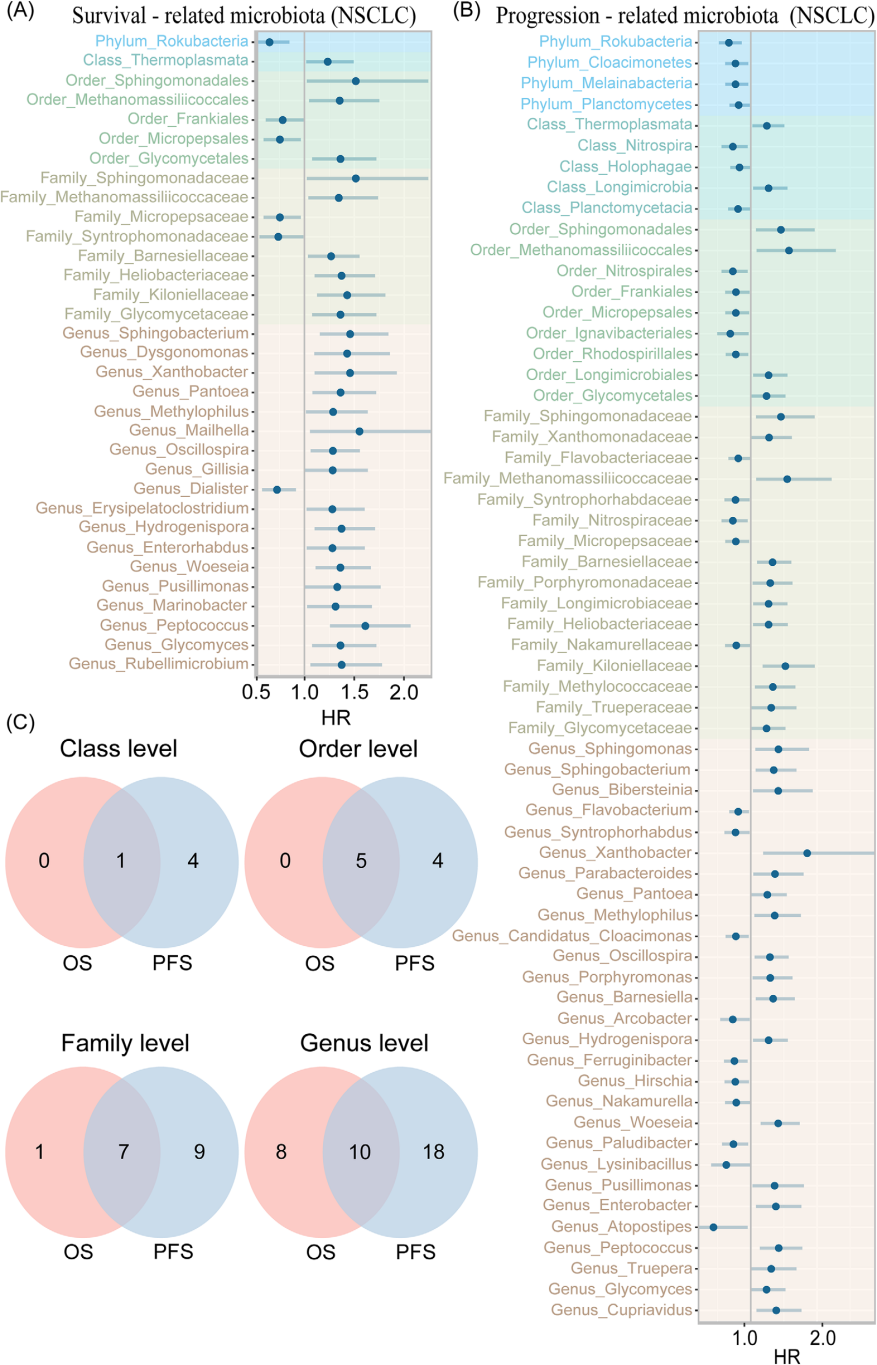
Screening prognosis‐related microbiota in NSCLC. (A and B) The prognosis screening of microbiota, at phylum, class, order, family and genus levels, related to OS and PFS, was conducted using univariate Cox regression (*p* < .05). HR > 1 and <1 represent risk and protective factors, respectively. (C) The Venn diagram shows compositional associations among microbiota.

We performed a prognosis‐related analysis of multi‐level microbiota using univariate Cox regression; one phylum, one class, five orders, eight families and 18 genera were significantly associated with OS, while four phyla, five classes, nine orders, 16 families and 28 genera were significantly related to PFS (Figure 4A,B). Therein, one phylum (*Rokubacteria*), one class (*Thermoplasmata*), five orders (*Sphingomonadales*, *Methanomassiliicoccales*, *Frankiales*, *Micropepsales*, *Glycomycetales*), seven families (*Sphingomonadaceae*, *Methanomassiliicoccaceae*, *Micropepsaceae*, *Barnesiellaceae*, *Heliobacteriaceae*, *Kiloniellaceae* and *Glycomycetaceae*) and 10 genera (*Sphingobacterium*, *Xanthobacter*, *Pantoea*, *Methylophilus*, *Oscillospira*, *Hydrogenispora*, *Woeseia*, *Pusillimonas*, *Peptococcus* and *Glycomyces*) were significantly related to both OS and PFS (Figure 4C). These microbiota also exhibited consistent beneficial or detrimental relationships with OS and PFS, further signifying significant role in NSCLC prognosis outcomes.

### 3.7 Functional analysis of prognosis‐related microbial clusters

**FIGURE 5:**
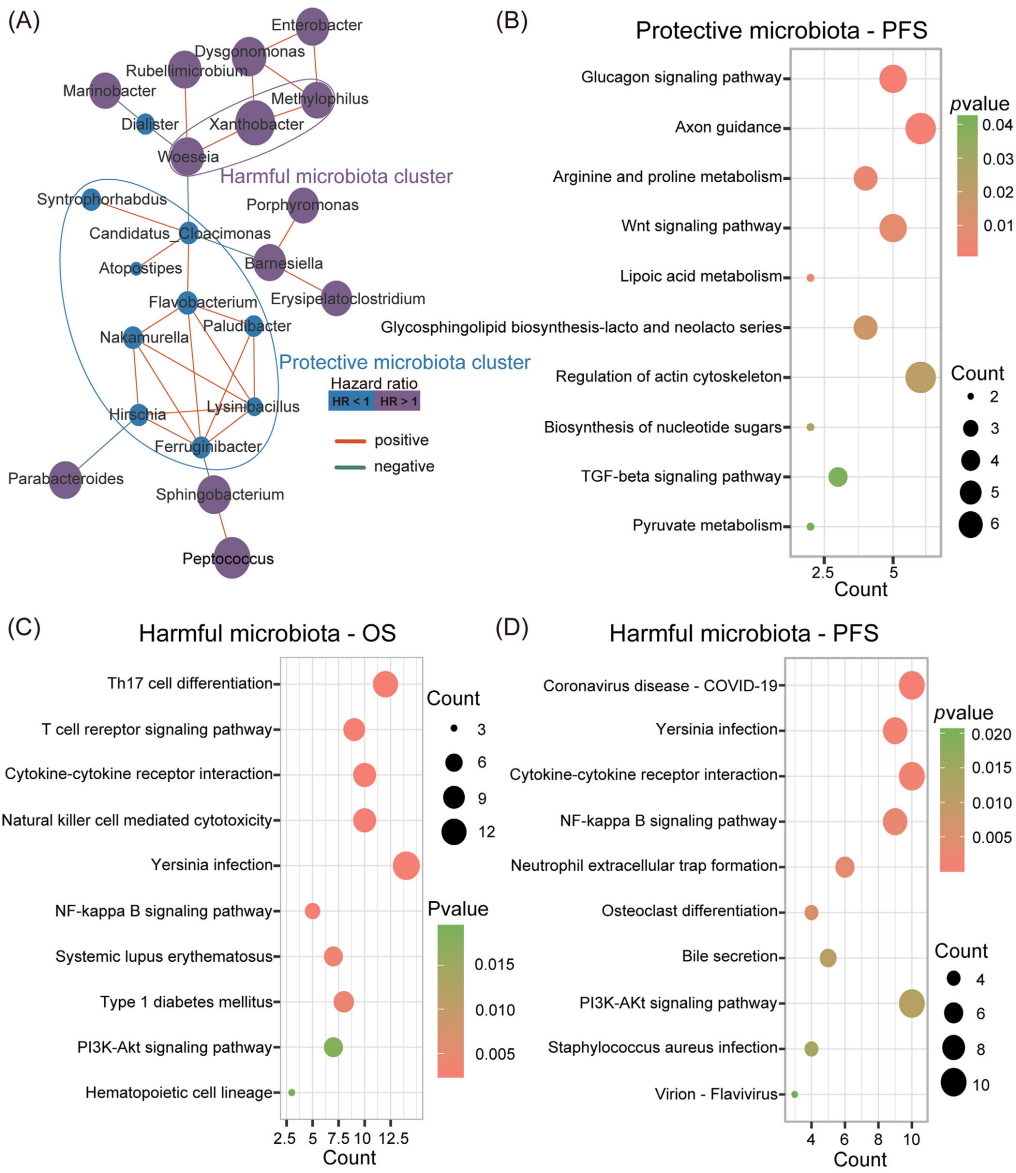
Analysis and functional prediction of prognostic microbiota clusters. (A) An ecological network showing prognostic microbial genera in NSCLC (SparCC, correlation > 0.4). (B–D) KEGG pathway enrichment analysis of genes associated with prognostic microbiota clusters.

We initially conducted a correlation analysis on 36 prognosis‐related microbial genera. After eliminating weak correlations, 23 genera were presented in the ecological network (Figure 5A). Of these, a positive correlation was identified among nine genera, all of which were associated with a longer PFS (HR < 1). Therefore, these nine genera were called the protective microbial cluster (PMC). We also discovered three positively correlated genera, all of which were associated with shorter OS and PFS times (HR > 1); therefore, these were known as the harmful microbial cluster (HMC).

To further explore potential cluster functions, we used univariate Cox regression and correlation analysis. The PMC was related to 214 PFS genes (*p* < .01) and the HMC was related to 186 OS genes (FDR < 0.05) and 153 PFS genes (*p* < .01, Tables S4–S6). After KEGG enrichment analyses, the PMC was mainly related to metabolic‐related pathways, including glucagon signalling, arginine and proline metabolism, lipoic acid metabolism, biosynthesis of nucleotide sugars and pyruvate metabolism (Figure 5B). The HMC was mainly related to immunity and inflammation (Th17 cell differentiation, T cell receptor signalling, cytokine–cytokine receptor interactions, nuclear factor‐kappa B (NF‐κB) signalling, natural killer cell‐mediated cytotoxicity, neutrophil extracellular trap formation), microbial infections (*Yersinia* infection and coronavirus disease—COVID‐19) and tumour‐related signalling (phosphatidylinositol 3‐kinase‐protein kinase B (Akt) signalling; Figure 5C,D). Putatively, the PMC mainly exerted protective effects via metabolic‐related functions, while the HMC mainly affected prognosis via infection invasion, thereby inducing changes in inflammation‐, immunity‐ and tumour‐related pathways.

### 3.8 Constructing a microbial prognostic model and analysing prognostic difference mechanisms

**FIGURE 6:**
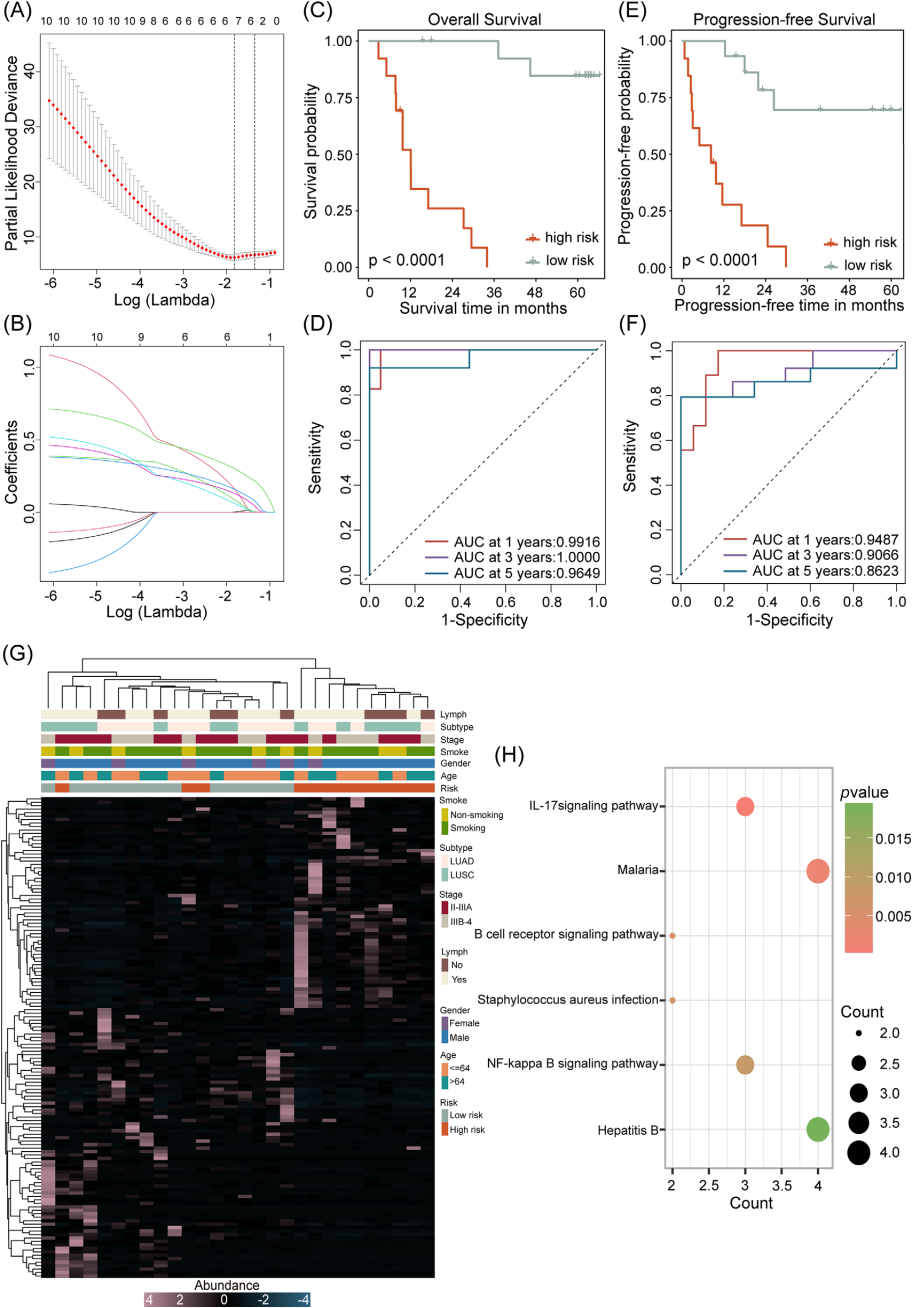
A prognostic model of microbial genera. (A) LASSO Cox analysis of microbial genera in our NSCLC cohort. The coefficient for each microbial genus associated with OS is represented as a curve. (B) The penalty parameter (*λ*) value was determined based on the minimum partial likelihood deviation using 10‐fold cross‐validation. (C and E) Kaplan–Meier survival curves showing OS and PFS differences between patients in high‐ and low‐risk groups. (D and F) Receiver operating characteristic (ROC) analyses predicting OS and PFS at 1, 3 and 5 years in patients. (G) Gene expression and clinical characteristic (gender, age, stage, subtype, smoking status and lymph node status) discrepancies between patients in high‐ and low‐risk groups. (H) KEGG pathway enrichment analysis showing the influence of the microbiota model on prognosis.

Ten genera showed correlations with both OS and PFS outcomes. From screening (LASSO and multivariate Cox regression analyses), six genera (*Xanthobacter*, *Pantoea*, *Oscillospira*, *Hydrogenispora*, *Peptococcus* and *Glycomyces*) were used to construct a prognostic model in a cohort of 28 NSCLC patients (Figure 6A,B and Table S7). The model accurately distinguished OS in patients (*p* < .0001), with AUC values for 1, 3 and 5 years = 0.9916, 1.0000 and 0.9649, respectively (Figure 6C,D). The model also forecasted PFS in patients (*p* < .0001), with AUC values for 1, 3 and 5 years = 0.9487, 0.9066 and 0.8623, respectively (Figure 6E,F). The model also performed well in predicting OS and PFS outcomes in patients with LUAD and LUSC (LUAD‐OS: *p* < .0001, LUSC‐OS: *p* = .0006, LUAD‐PFS: *p* = .0001, LUSC‐PFS: *p* = .0020; Figure S2). Therefore, our model, composed of six genera, showed good predictive accuracy for NSCLC prognoses in patients.

In comparing high and low‐risk groups, no significant disparity was identified in alpha and beta diversity indices. Alpha diversity in the low‐risk group was marginally higher than in the high‐risk group (Figure S3A,B), suggesting that patients with a poorer prognosis may have had a more detrimental TME. This unfavourable TME may not be suitable for the survival of certain microbiota. And we hypothesise that the TME may play an active role in screening intratumoural microbiota. Hence, the microbiota may potentially function as a valuable and novel biomarker. Additionally, correlation analyses showed that among the six genera (all detrimental factors), *Hydrogenispora* and *Pantoea*, *Peptococcus* and *Xanthobacter*, and *Oscillospira* and *Glycomyces* exhibited tighter correlations (Figure S3C). These genera were considerably prevalent in the high‐risk group, in accordance with prognosis results (Figure S3D).

To further explore if the microbiota affected tumour gene expression, we performed DEG analyses and observed distinct gene expression profiles between high and low‐risk groups. Of these, 82 genes were up‐regulated in the low‐risk group and 63 up‐regulated in the high‐risk group (FDR < 0.05 and |LogFC| > 1; Figure 6G). To further determine potential mechanisms, a correlation analysis was performed between the six genera and DEGs, with ultimately 37 related genes identified. KEGG enrichment results demonstrated that infections (malaria, *Staphylococcus aureus* and Hepatitis B), inflammation (interleukin (IL)‐17 and NF‐κB signalling) and immune regulation (B cell receptor signalling) were the principal pathways whereby the microbiota affected prognosis outcomes (Figure 6H). This was analogous to HMC prognosis‐related pathways. Thus, the microbiota may impact prognosis outcomes by causing infection and inducing inflammation and immune responses.

### 3.9 The *Peptococcus* genus may affect prognosis via tumour necrosis factor signalling

**FIGURE 7:**
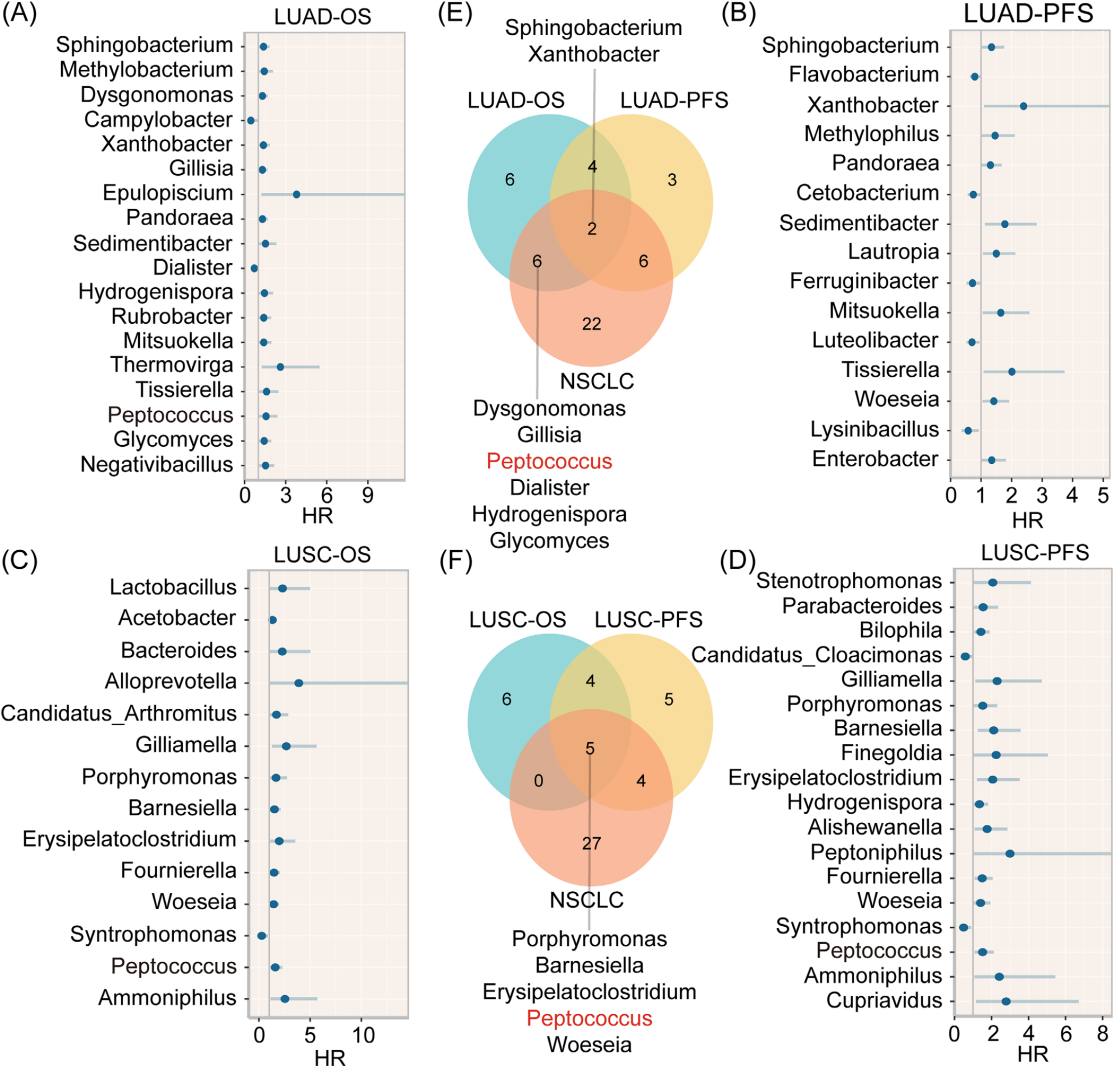
Screening prognosis‐related microbial genera in LUAD and LUSC. (A–D) The prognosis screening of microbial genera related to OS and PFS in LUAD and LUSC was conducted using univariate Cox regression (*p* < .05). HR > 1 and <1 represent risk and protective factors, respectively. (E and F) The Venn diagram shows compositional associations among prognostic microbial genera.

Given that LUAD and LUSC microbial composition varied, we separately conducted a prognostic screen of microbial genera in LUAD and LUSC (Figure 7A–D), which showed compositional disparities in prognosis‐related microbial genera across tissue types. In particular, *Peptococcus* shows the most positive results in terms of prognosis for NSCLC, LUAD and LUSC (*p* < .05; Figure 7E). Also, *Peptococcus* levels in the high‐risk group were significantly higher than those in the low‐risk group (*p* < .01; Figure S3D). Hence, *Peptococcus* showed a significant association with a poorer prognosis.

**FIGURE 8:**
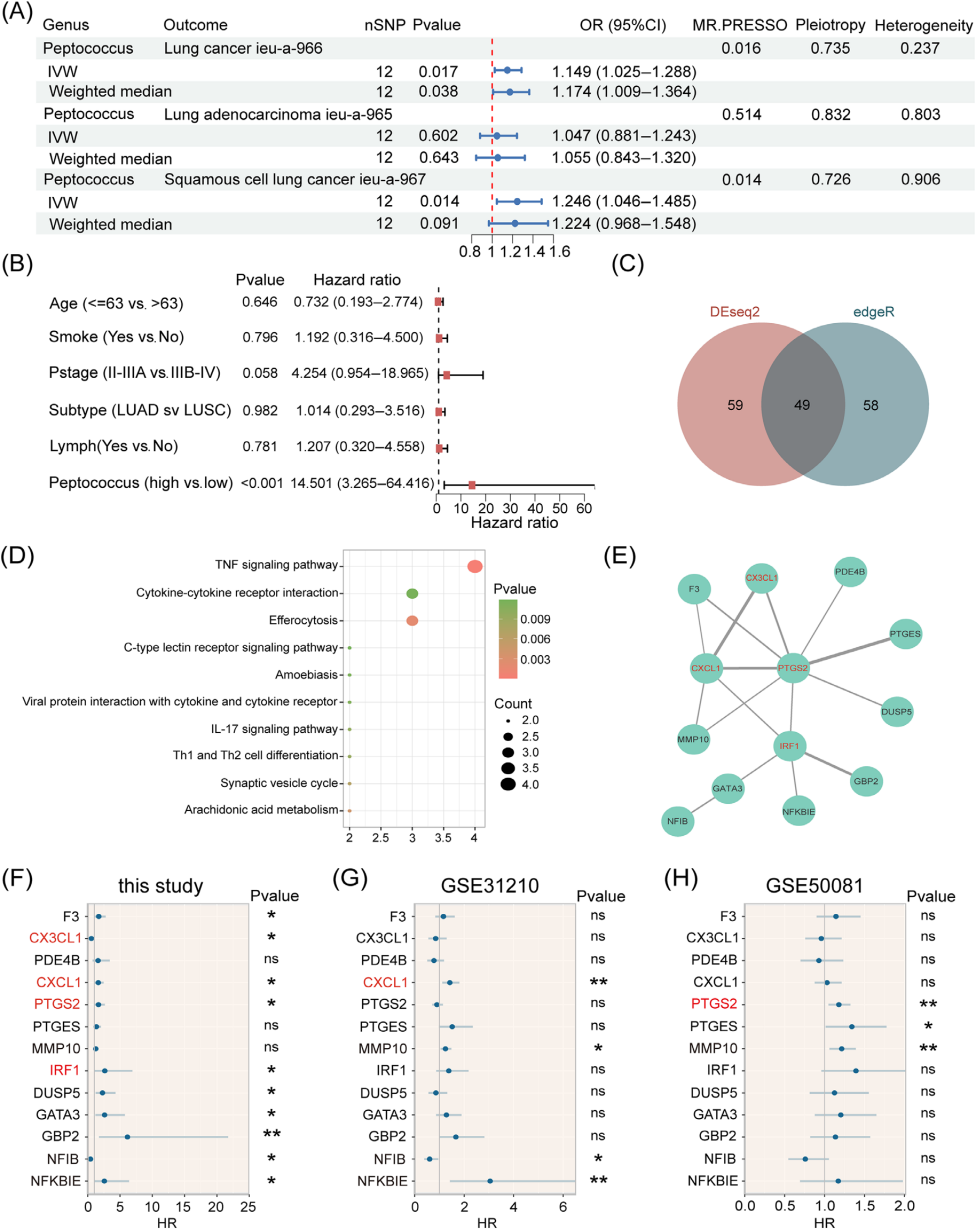
Potential mechanisms underpinning *Peptococcus* effects on prognosis. (A) Mendelian randomisation was used to test for causal relationships between *Peptococcus*, lung cancer, LUAD and LUSC using inverse‐variance weighted, weighted median and MR. PRESSO methods. When no heterogeneity and pleiotropy occurred (*p* > .05), and at least one test method yielded a positive result (*p* < .05), a positive causal relationship existed between exposure and outcome. (B) Multivariate Cox regression analysis was used to test whether the *Peptococcus* influence on prognosis was independent of common clinical factors (age, smoking status, stage, subtype and lymph node status). (C) DESeq2 and edgeR were used to screen DEGs between groups which were divided based on *Peptococcus* levels in patients (FDR < 0.05). Forty‐nine DEGs exhibited statistical discrepancies in both methods and were designated as common DEGs. (D) KEGG pathway enrichment analysis of 49 common DEGs. (E) The network of 13 core gene products screened from 49 gene products (corresponding to common DEGs) through protein–protein interaction network (PPI) (interaction score > 0.4). Genes in red represent those from the TNF signalling pathway. (F–H) Univariate Cox regression analysis of the prognostic relationships of the 13 core genes, GSE31210 and GSE50081 datasets. NS > 0.05; *p < .05; **p < .01; ***p < .001.

Using MR, we identified a significant causal relationship between *Peptococcus* and lung cancer (*p* = .017, odds ratio (OR) = 1.149) and LUSC (*p* = .014, OR = 1.246), but no relationship with LUAD (*p* = .602, OR = 1.047; Figure 8A). Similarly, in a reverse MR analysis, no causal relationship was identified between lung cancer and *Peptococcus*. The results of the MR analysis indicate that *Peptococcus* may have a promoting effect on NSCLC, but NSCLC has no influence on *Peptococcus*. Therefore, *Peptococcus* is very likely a new genus that affects NSCLC prognosis outcomes.

To explore potential pathways whereby *Peptococcus* influenced prognosis, we divided 28 patients into high‐abundance (abundance > the median value) and low‐abundance groups (abundance ≤ the median value) based on *Peptococcus* levels. In a prognostic multivariate Cox regression analysis of these levels together with age, smoking, stage, subtype and lymph node status, *Peptococcus* was identified as an independent prognostic factor (Figure 8B). We then used DESeq2 and edgeR methods to identify 108 and 107 DEGs between groups, respectively, of which 49 were common DEGs (Figure 8C). After KEGG enrichment analysis, Tumour necrosis factor (TNF) signalling was the main enriched pathway (Figure 8D). We then conducted correlative analyses between enriched genes in the TNF pathway and *Peptococcus* abundance and found that *Peptococcus* exhibited a marked positive correlation with *C‐X‐C motif chemokine ligand 1* (*CXCL1*, *R* = 0.46, *p *= .013), *Interferon regulatory factor 1* (*IRF1*, *R* = 0.51, *p *= .0056) and *Prostaglandin‐endoperoxide synthase 2* (*PTGS2*, *R* = 0.50, *p* = .0068), and a substantially negative correlation with *C‐X3‐C motif chemokine ligand 1* (*CX3CL1*, *R* = −0.49, *p* = .0086; Figure S4). These observations suggested that TNF signalling may be a pathway whereby *Peptococcus* impacts on prognosis outcomes. We also identified 13 core gene products using the protein–protein interaction (PPI) network, with aforementioned gene products showing core positions in this network (Figure 8E). Next, in univariate Cox regression analysis, these genes were associated with prognosis outcomes (*CXCL1*: HR = 1.63, 95% confidence interval (CI): 1.08–2.45, *p* < .05; *IRF1*: HR = 2.63, 95% CI: 1.00–6.90, *p* < .05; *PTGS2*: HR = 1.65, 95% CI: 1.04–2.64, *p* < .05 and *CX3CL1*: HR = 0.56, 95% CI: 0.33–0.93, *p* < .05; Figure 8F).

Considering that most patients were in stages II–IV, we also conducted the same analysis in two stages I–II NSCLC datasets and found that *CXCL1* was significantly associated with prognosis in the GSE31210 dataset (HR = 1.42, 95% CI: 1.12–1.81, *p* < .01) and *PTGS2* in the GSE50081 dataset (HR = 1.18, 95% CI: 1.05–1.32, *p* < .01; Figure 8G,H). Thus, *Peptococcus* may lead to an unfavourable prognosis in NSCLC patients by influencing TNF signalling.

## 4 DISCUSSION

**FIGURE 9:**
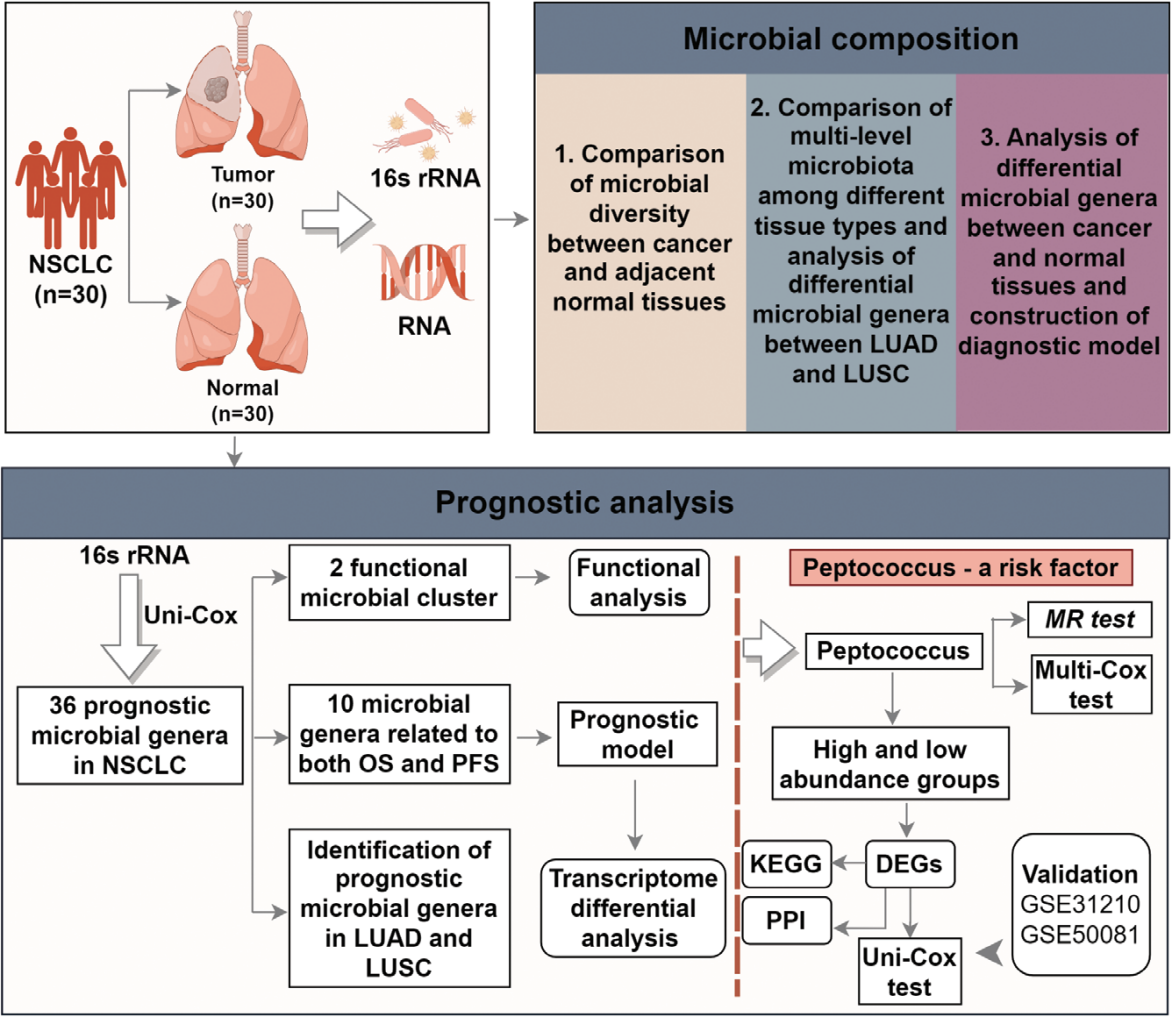
Study workflow (assembled in Figdraw). NSCLC, non‐small cell lung cancer; LUAD, lung adenocarcinoma; LUSC, lung squamous cell carcinoma; uni‐Cox, univariate Cox regression; multi‐Cox, multivariate Cox regression; OS, overall survival; PFS, progression‐free survival; MR, mendelian randomisation; DEGs, differentially expressed genes; KEGG, Kyoto Encyclopedia of Genes and Genomes; PPI, protein–protein interactions.

Previous studies have reported that the microbiota is intimately associated with cancer, with some species showing causal connections with tumourigenesis, progression and metastasis, especially in the digestive system. Critically, microbiota research in NSCLC is at an early stage, with insufficient evidence linking specific microbiota to the disease. Therefore, identifying key microbiota and compositional characteristics in NSCLC may help refine clinical management approaches. In this study, we performed *16S rRNA* and transcriptome sequencing on 30 NSCLC and adjacent tissue pairs and recorded prognostic information from patients. Microbial composition in NSCLC differed to that in normal tissues, with dissimilarities between LUAD and LUSC. More importantly, we identified four phyla, five classes, nine orders, 17 families and 36 genera that were significantly related to disease prognosis. Of the 36 genera, a PMC (nine genera) was associated with a longer PFS and suggested relationships with metabolic functions. Conversely, a HMC (three microbiota) was associated with shorter OS and PFS and suggested relationships with infection, inflammation and immune pathways. Additionally, *Peptococcus* was identified as an independent risk factor for a poor prognosis in NSCLC patients, and possibly mediated its effects via TNF signalling. These findings suggested a critical role for intratumoural microbiota in NSCLC pathogenesis and may provide novel perspectives for future research. The flowchart of this study is presented in Figure 9.

Between NSCLC and paired tissues, no significant alpha diversity disparity was observed, consistent with previous research^27^; diversity in NSCLC was marginally higher than in normal tissues. However, other studies have also found that the alpha diversity of early‐stage LUAD is significantly higher than that of the matched normal lung tissues.^28^ The comparison results of this alpha diversity are different from those of this study. Therefore, this phenomenon still requires more research to be confirmed. Previous studies have reported that bacterial quantities in different tumour tissues, such as breast cancer and ovarian cancer,^3^ are significantly higher than in normal tissues. However, this does not suggest no variance in microbial composition between NSCLC and normal tissues, as communications between normal lung tissues and the exterior must be considered. When examining beta diversity among different tissue types, only LUSC exhibited more pronounced microbial differences when compared with normal tissues. However, whether this phenomenon is associated with the distinctive LUSC microenvironment remains unknown.

We compared multi‐microbiota levels across tissue types and discovered that microbial composition in LUAD and LUSC was disparate. Likewise, in microbial analyses of LUAD and LUSC, we identified numerous, differentially abundant microbial genera, and we perceived that the correlation of these differential genera in LUAD and LUSC were dissimilar. These observations were analogous to previous research findings^29^ and may contribute to future microbial research in NSCLC.

In differential analyses between cancer and normal tissues, we identified 12 markedly differential genera. Of these, *Methylobacterium* was previously associated with adverse prognosis outcomes in gastric cancer.^30^ Additionally, two probiotics (*Lactobacillus* and *Akkermansia*) were also enriched in NSCLC tissues. Previous studies have reported that *Lactobacillus* restrained tumour cell metastasis to the lungs and showed anti‐tumour activity in in vitro experiments.^31^, ^32^ *A. muciniphila* also augmented the anti‐tumour effects of cisplatin in Lewis lung cancer mice and was potentially related to effective immunotherapy.^33^, ^34^ While these microbiota showed correlations with cancer, their role in NSCLC remains ambiguous; therefore, future studies must verify these observations.

Upon screening, 36 genera showed correlations with NSCLC prognosis outcomes, from which 10 were related to cancer. Previous studies have indicated that *Porphyromonas* is more prevalent in the gut or tumour tissues of patients with gastrointestinal cancers,^35^, ^36^ with *Porphyromonas gingivalis* abundance a crucial risk determinant for oesophageal squamous cell carcinoma.^37^ It is noteworthy that Ni and colleagues also identified *Marinobacter* in early‐stage LUAD,^38^ but *Marinobacter* functions in NSCLC are unknown. Additionally, multiple studies have reported positive correlations between *Oscillospira* and gastric cancer,^39^ *Erysipelatoclostridium* and oral cancer,^40^ *Sphingomonas* and thyroid cancer^41^ and *Enterobacter* and ovarian cancer,^42^ consistent with our results (HR > 1). Beyond harmful microbial genera (HR > 1), we also observed that *Lysinibacillus* was associated with longer PFS, with two studies reporting that a *Lysinibacillus sphaericus* toxin exerted anti‐tumour effects in multiple tumour cell lines.^43^, ^44^ Therefore, *Lysinibacillus* in NSCLC requires more investigation. We also identified phenomena contrary to previous research; for instance, *Dialister* was related to a favourable prognosis in our study, but Lyu and colleagues^45^ reported that *Dialister* was related to oral squamous cell carcinoma recurrence. Similarly, *Parabacteroides* was associated with early recurrence in our study, but elsewhere, *Parabacteroides distasonis* enhanced immunotherapy responses in bladder cancer.^46^ Furthermore, all the other 26 prognosis‐related microbial genera are proposed to be related to tumours for the first time.

Then, we conducted functional predictions in two microbiota clusters. Analogous to prognostic model functional outcomes, the PMC was primarily associated with metabolism‐related pathways, while the HMC was mainly linked to infection, immunity and inflammation pathways. These results largely concurred with previous gut microbiota and cancer research. Currently, microbial metabolites mainly include secondary bile acids, short‐chain fatty acids (SCFAs), polyamines and tryptophan and associated derivatives.^47^ Some studies have indicated that tryptophan metabolites, conjugated linoleic acids, SCFAs and polyphenolic metabolites can prevent CRC development,^35^, ^48^, ^49^ while sodium butyrate exerts anti‐tumour activity against breast cancer.^50^ Other studies reported that the microbiota was closely related to immunity and inflammation; e.g., *Fusobacterium nucleatum* facilitated *IL‐8* and *CXCL1* excretion, thereby inhibiting HCT116 (human colorectal carcinoma) cell proliferation and migration.^51^ Additionally, the microbiota also promoted tumourigenesis and disease progression by directly invading or secreting virulence factors to cause DNA damage; for example, *Salmonella typhi* secreted various virulence factors and caused DNA damage and inflammation.^52^ Therefore, we hypothesise that intratumoural microbiota functions are analogous to the gut microbiota. In general, we discovered protective and harmful microbiota in NSCLC tumours; the PMC had a lesser impact on immunity and mainly exerted anti‐tumour effects via metabolism‐related pathways, while the HMC mainly facilitated tumour progression via cell invasion and induced inflammatory and immune responses.

We developed a prognostic model utilising 10 microbial genera that are associated with both OS and PFS. This model exhibits high accuracy in determining the survival and recurrence status of patients within 5 years. This provides a novel biomarker for the prognostic stratified management of NSCLC patients. In our analysis, many microbiota appear as harmful or beneficial factors for tumours. At the same time, we also observe a trend of differences in microbial diversity among patients with different prognoses (high and low risk). This indicates that the interaction between microbiota and tumours is mutual. Previous studies have shown that cancer can control the central neuroendocrine and immune systems and reset the body's homeostasis to favour its expansion at the expense of the host.^53^ Prognosis may represent differences in the local TME or even the body's homeostasis, which may directly affect the characteristics of intratumoural microbial composition. Conversely, certain microbiota may promote or inhibit tumour occurrence and development in multiple ways, including but not limited to their metabolites.^54^ In short, a comprehensive understanding of the complex relationship between microbiota and tumours is essential for future evidence‐based and holistic treatment approaches.

We also observed that *Peptococcus* was a principal independent prognostic factor for NSCLC, with its occurrence potentially meaning a poor prognosis for patients. In a previous study, considerable *Peptococcus* levels were identified in oral squamous cell carcinoma^55^; however, *Peptococcus* actions in tumours remain unclear. To address this, we performed a functional analysis which showed that TNF signalling may be the principal pathway through which *Peptococcus* influences prognosis, with *Peptococcus* significantly and positively correlated with *CXCL1*, *PTGS2* and *IRF1* TNF‐pathway components. From the literature, we observed that *Peptostreptococcus anaerobius* interacted with integrin α2β1 in CRC and promoted *CXCL1* secretion by activating NF‐κB, with the latter interacting with *C‐X‐C chemokine receptor type 2* (*CXCR2*) on myeloid‐derived suppressor cells (MDSCs), thereby promoting MDSC migration, reducing functional T cell infiltration and creating an immunosuppressive microenvironment conducive to CRC.^56^ Another study reported that Gram‐negative bacteria converted arachidonic acid to prostaglandin E2 by up‐regulating PTGS2 or PTGES expression and promoting CRC cell proliferation.^57^ Intriguingly, both *Peptococcus* and *Peptostreptococcus* genera were affiliated with *Clostridiales* and featured similar structures, thereby providing evidence for subsequent studies. Additionally, Harioudh and colleagues^58^ showed that IRF1 exerted antibacterial protective effects. Combined, these research results suggested tight linkage between *CXCL1, PTGS2, IRF1* and the microbiota, with these genes potentially triggering immune and inflammatory responses. Thus, *Peptococcus* may be a ‘novel’, crucial microbiota in NSCLC and impact on patient prognoses by regulating inflammatory and immune environments.

This study is admittedly subject to certain limitations. First, it must be acknowledged that this is a small‐scale omics research. Additionally, in this paper, only GWAS and RNA data are employed as the external validation dataset, without including an external microbial validation cohort. These results have not been verified in vitro and in vivo experiments. Furthermore, there are numerous factors that can influence patient prognosis, such as tumour mutations, postoperative treatment and other diseases. These factors are not taken into consideration in the prognostic analysis of this study.

## 5 CONCLUSIONS

We identified notable correlations between intratumoural microbiota and prognosis outcomes in NSCLC patients. A PMC mainly performed metabolic functions, while a HMC mainly exerted infection, inflammation and immune modulation effects. Moreover, *Peptococcus* may have a crucial role in adverse NSCLC prognoses in patients and serve as a biomarker for patient management and cancer screening.

## AUTHOR CONTRIBUTIONS

Jun Chen is responsible for the overall content as the guarantor. Fuling Mao, Ruifeng Shi, Zixuan Hu, Yongwen Li, Hongyu Liu and Jun Chen designed the experiments. Fuling Mao, Ruifeng Shi, Hongbing Zhang, Zihe Zhang, Xuguang Li, Minghui Liu, Penghu Gao, Zhanrui Zhang, Yingjie Wang and Dikai Ding collected clinical samples and performed the related experiments. Fuling Mao, Ruifeng Shi, Zixuan Hu and Jinhui Li analysed the data. Fuling Mao, Hongyu Liu and Jun Chen wrote the manuscript. All authors edited the manuscript.

## CONFLICT OF INTEREST STATEMENT

The authors declare no conflict of interest.

## ETHICS STATEMENT

Ethical approval was obtained from the Institutional Review Board of Tianjin Medical University General Hospital (ID: IRB2023‐YX‐092‐01). All patients provided written informed consent. The study was conducted in accordance with the principles stated in the Declaration of Helsinki.

## Supporting information



Supporting Information

Supporting Information

Supporting Information

Supporting Information

Supporting Information

Supporting Information

## Data Availability

The datasets (GSE31210 and GSE50081) for this study can be found in the GEO Datasets (https://www.ncbi.nlm.nih.gov). The *16S rRNA* and transcriptome sequencing data have been deposited in the Genome Sequence Archive in National Genomics Data Center, China National Center for Bioinformation, Chinese Academy of Sciences (OMIX: OMIX007164 and GSA‐Human: HRA006222) that are publicly accessible at https://ngdc.cncb.ac.cn/gsa‐human. The other data can be obtained from the corresponding author upon reasonable request.
