# Supplementary figures and images for: Unravelling the prognostic and operative role of intratumoural microbiota in non‐small cell lung cancer: Insights from 16S rRNA and RNA sequencing
Source: Clin Transl Med. 2025 Jan 3;15(1):e70156. doi: 10.1002/ctm2.70156 (PMC11702424; doi:10.1002/ctm2.70156)

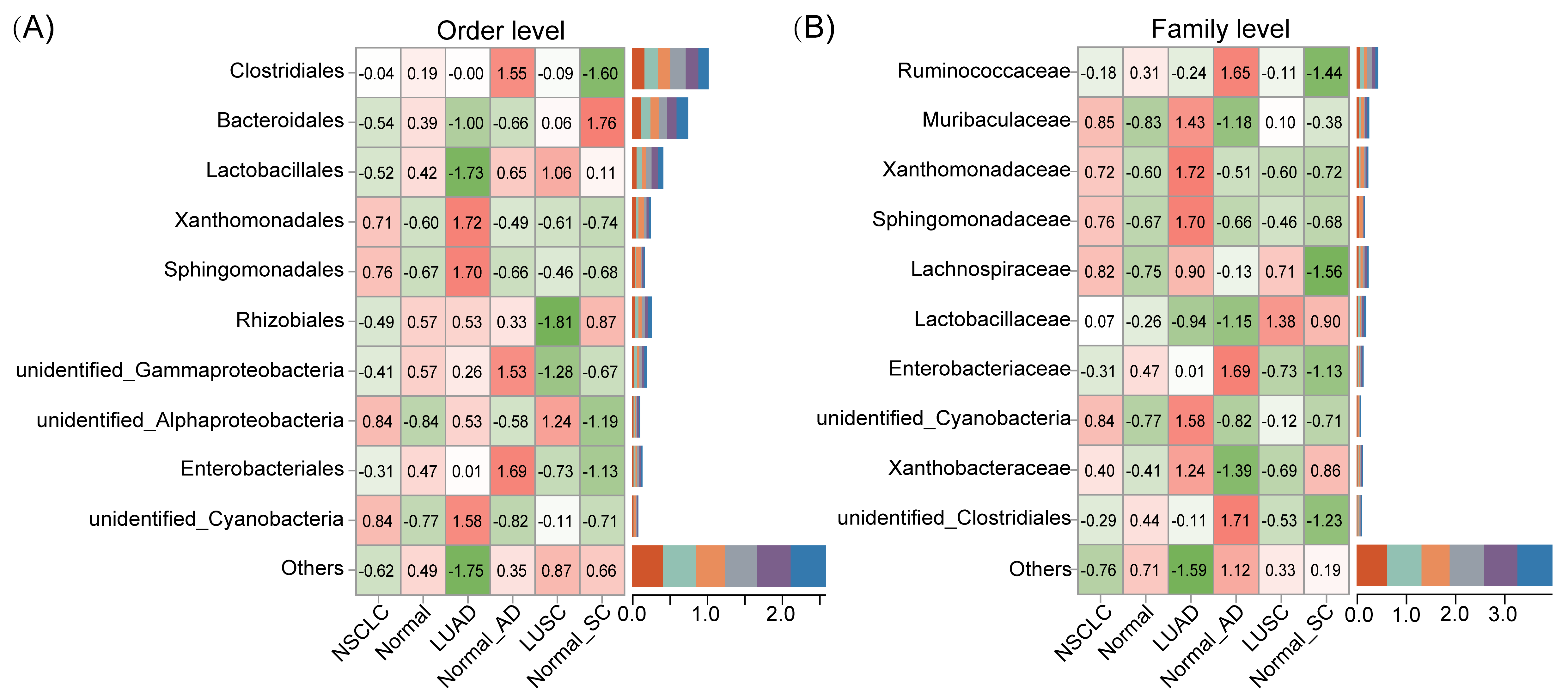

Supplement: Supplementary file 1 — Supporting Information [file CTM2-15-e70156-s003.tif]

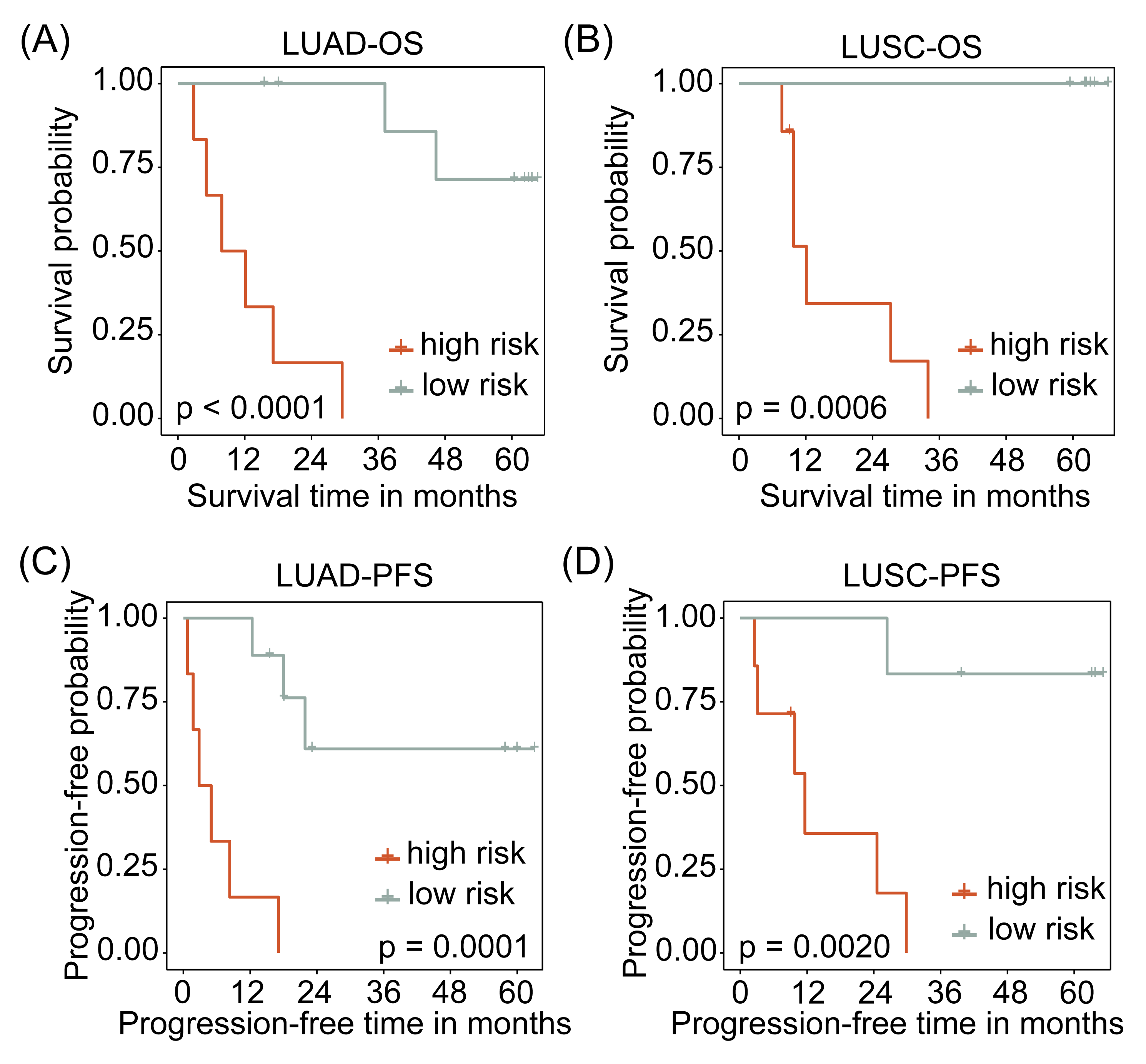

Supplement: Supplementary file 2 — Supporting Information [file CTM2-15-e70156-s002.tif]

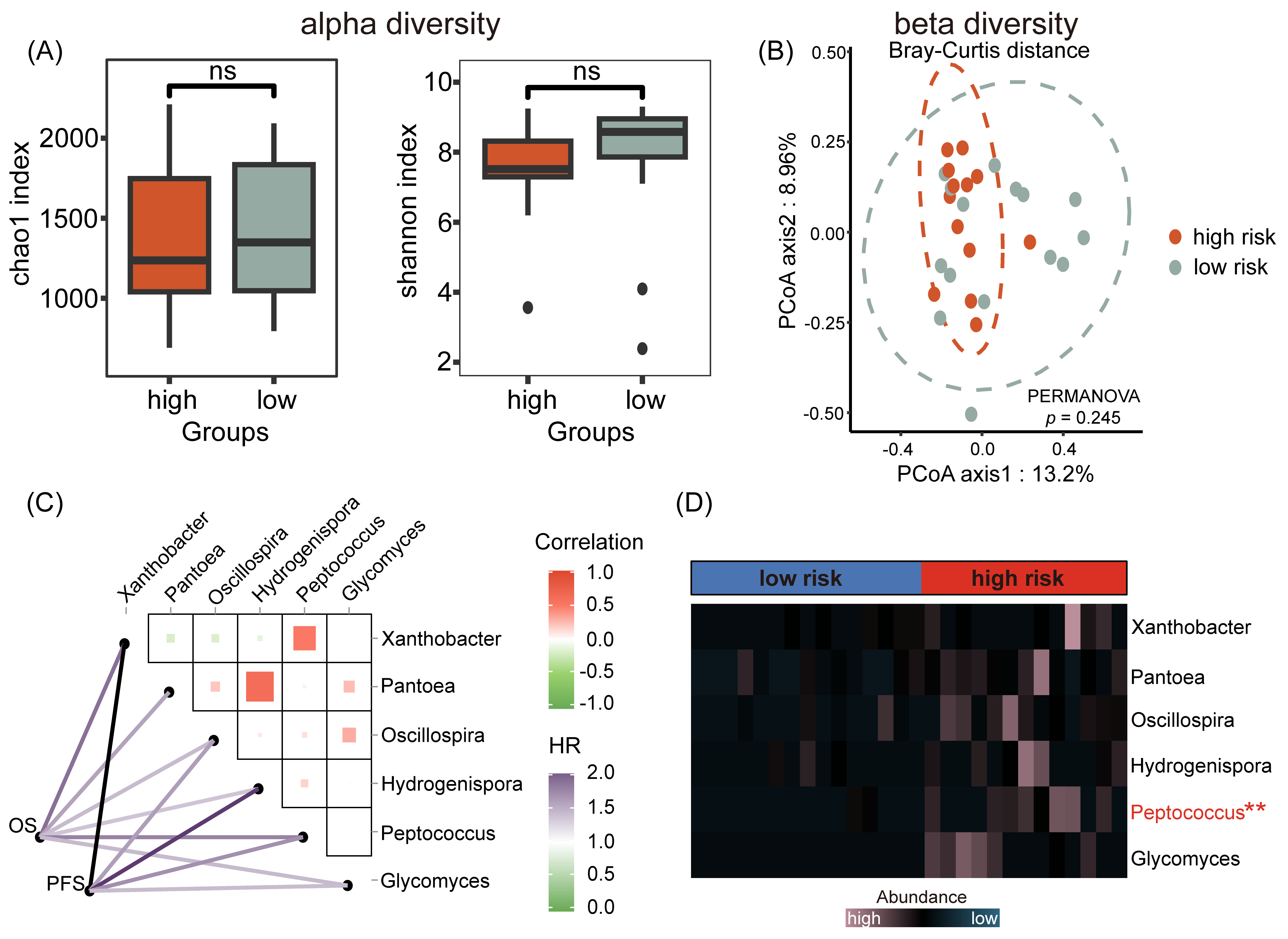

Supplement: Supplementary file 3 — Supporting Information [file CTM2-15-e70156-s001.tif]

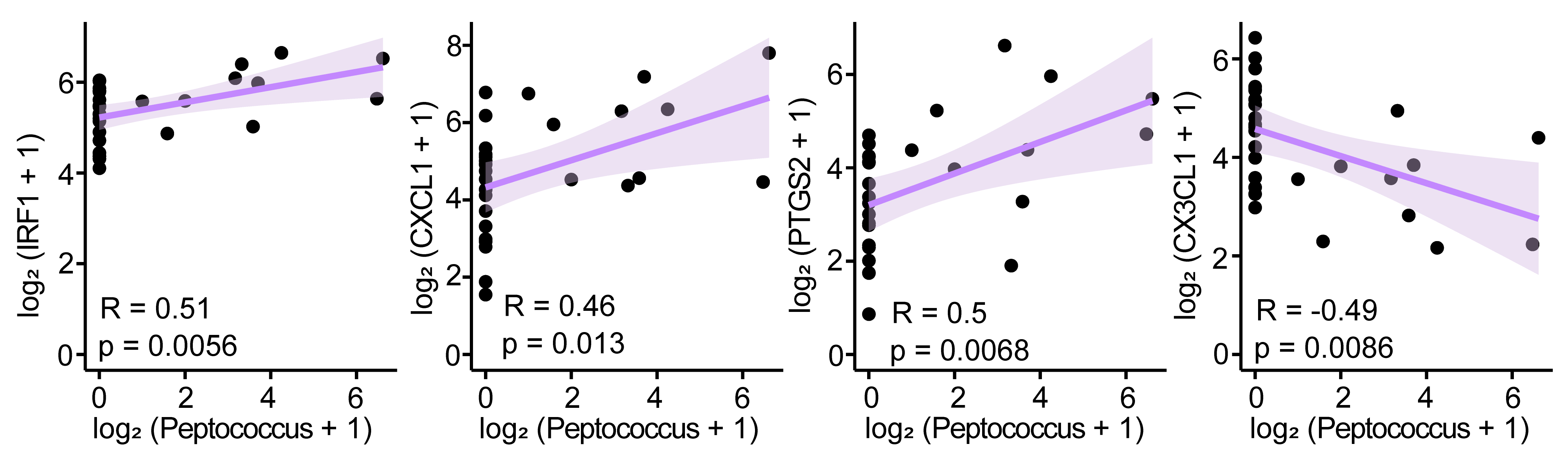

Supplement: Supplementary file 4 — Supporting Information [file CTM2-15-e70156-s005.tif]
