# Supplementary material for: Unravelling the prognostic and operative role of intratumoural microbiota in non‐small cell lung cancer: Insights from 16S rRNA and RNA sequencing
Source: Clin Transl Med. 2025 Jan 3;15(1):e70156. doi: 10.1002/ctm2.70156 (PMC11702424; doi:10.1002/ctm2.70156)
Supplement: Supplementary file 6 — Supporting Information [file CTM2-15-e70156-s004.docx]

**Figure S1** Analysis of the composition differences of microbiota at the Order and Family levels in different types of lung cancer tissues and corresponding adjacent tissues. The top 10 microbiota at each classification level were defined as high-abundance microbiota, and the other microbiota were referred to as low-abundance microbiota. The values in the heatmap (left panel) are the abundance values normalized by row. The barplot (right panel) is used to show the proportion of a single microbiota in each group of samples.

**Figure S2** Survival curve analysis of the microbial prognostic model for predicting the prognosis (OS and PFS) of LUAD and LUSC patients. Red represents a higher risk of poor prognosis for patients, and green represents a lower risk of poor prognosis for patients. The chi-square test was used to test the prognostic differences between the two groups.

**Figure S3** Analysis of microbial differences between high and low-risk groups. (A) Difference analysis of alpha diversity (chao1 and shannon indices) between high and low-risk groups. ^ns^P >.05; *P <.05; **P <.01; ***P <.001. (B) The Permutational Multivariate Analysis of Variance (PERMANOVA) test was used to compare the beta diversity between high- and low-risk groups (calculated based on Bray-Curtis distance). Principal Co-ordinates Analysis (PCoA) was used for the visualization of data dimensionality reduction analysis. (C) Correlation analysis between the six microbial genera in the prognostic model and OS and PFS through the Pearson method. (D) Heatmap of the abundance levels of six microbial genera between high and low-risk groups. The abundance values have been standardized and normalized.

**Figure S4** Correlation analysis between genus *Peptococcus* and genes of the TNF signaling pathway. The levels of genus *Peptococcus* and each gene have been standardized, and Pearson was used for the correlation analysis.

**Table S1 and S2** The clinical characteristics of 30 NSCLC patients.

**Table S3** Details of the diagnostic model composed of 5 microbiotas.

**Table S4** The relevant information of the PFS related genes of the protective microbiota cluster (*p* < 0.01).

**Table S5** The relevant information of the OS related genes of the harmful microbiota cluster (FDR < 0.05).

**Table S6** The relevant information of the PFS related genes of the harmful microbiota cluster (*p* < 0.01).

**Table S7** Details of the prognostic model composed of 6 microbiotas.
